# The Effectiveness of Educational Interventions on Breast Cancer Screening Uptake, Knowledge, and Beliefs among Women: A Systematic Review

**DOI:** 10.3390/ijerph18010263

**Published:** 2020-12-31

**Authors:** Sarah Noman, Hayati Kadir Shahar, Hejar Abdul Rahman, Suriani Ismail, Musheer Abdulwahid Al-Jaberi, Meram Azzani

**Affiliations:** 1 Department of Community Health, Faculty of Medicine and Health Sciences, Universiti Putra Malaysia, Serdang 43400, Malaysia; saranoman12@gmail.com (S.N.); hejar@upm.edu.my (H.A.R.); si_suriani@upm.edu.my (S.I.); musheer.jaberi@gmail.com (M.A.A.-J.); 2 Malaysian Research Institute of Ageing (MyAgeing), Serdang 43400, Malaysia; 3 Community Medicine Department, Faculty of Medicine, MAHSA University, Saujana Putra Campus, Jenjarom 42610, Malaysia; dr_memeazzani@yahoo.com

**Keywords:** breast cancer, breast cancer screening, educational interventions, knowledge, beliefs

## Abstract

There have been various systematic reviews on the significance of educational interventions as necessary components to encourage breast cancer screening (BCS) and reduce the burden of breast cancer (BC). However, only a few studies have attempted to examine these educational interventions comprehensively. This review paper aimed to systematically evaluate the effectiveness of various educational interventions in improving BCS uptake, knowledge, and beliefs among women in different parts of the world. Following the PRISMA guidelines, a comprehensive literature search on four electronic databases, specifically PubMed, Scopus, Web of Science, and ScienceDirect, was performed in May 2019. A total of 22 interventional studies were reviewed. Theory- and language-based multiple intervention strategies, which were mainly performed in community and healthcare settings, were the commonly shared characteristics of the educational interventions. Most of these studies on the effectiveness of interventions showed favorable outcomes in terms of the BCS uptake, knowledge, and beliefs among women. Educational interventions potentially increase BCS among women. The interpretation of the reported findings should be treated with caution due to the heterogeneity of the studies in terms of the characteristics of the participants, research designs, intervention strategies, and outcome measures.

## 1. Introduction

The burden of breast cancer (BC) is enormous. Approximately 2.1 million female BC cases were diagnosed in 2018 alone. Apart from being the most diagnosed cancer globally, BC is believed to be the leading reason of cancer mortality in over 100 countries [1]. The mortality rates of BC have increased in certain countries which have historically had a lower rate, such as sub-Saharan Africa [1]. This trend is probably due to the increase in the incidence rates of BC with limited access to early detection and treatment, and late-stage diagnosis [2,3].

Early detection of the disease can save lives and increase the chances of being treated efficaciously [4]. The diagnosis of BC during the local stage (stage I and some stage II) has an overall 5-year relative survival rate of 99% while the diagnosis of BC during the regional stage (stage II or III) has a 5-year relative survival rate of 85%. However, the late stage (some stage III and all stage IV) diagnosis of BC has an overall 5-year relative survival rate of 27% [5]. Numerous methods have been assessed as breast cancer screening (BCS) approaches, such as breast self-examination (BSE), clinical breast examination (CBE), and mammography (MMG) [6]. Getting regular screening examinations is the key strategy for detecting BC early and reducing the mortality rate of the disease [7]. However, a number of epidemiological studies on BCS behavioral uptake have been performed on community samples of various groups of women. Such studies have shown that the rate of BCS practice is low in various countries [8,9,10,11,12,13].

A good knowledge of BC and BCS is a prerequisite for the adherence to BCS among women [14]. A low level of knowledge among women in different parts of the world is linked to a low practice rate of BCS [8,15,16,17,18,19]. Various international studies and guidelines have stressed the significance of educational interventions as necessary components of effective BCS programs in order to encourage BCS and reduce the burden of the disease among women [8,17,18,20,21,22,23].

Demographic factors such as age, socio-economic status, and ethnicity are known to be associated with the use of health services and preventive health behaviors [24]. However, health education cannot modify demographic factors. Hence, developing efficient health education targeting modifiable individual characters is a challenge that in turn can predict preventive service usage and health behavior. Since beliefs are influenced by individual characteristics, they could provide an ideal target, as they affect behavior and are possibly modifiable. Beliefs distinguish individuals from the same background. However, at the same time, beliefs may reflect different socialization histories arising from demographic variation [25].

People’s participation in health promotion programs is predicted using health behavior theories [26]. Through such theories, remarkable progress in studying the determinants of a person’s health-related behavior in developing positive changes has been achieved [27]. Breast cancer screening is one behavior that is influenced by women’s health beliefs [28]. This behavior has been explained in studies conducted in accordance with a variety of theories. In such studies, the meaning of BC and BCS were described by one concept related to one behavior, or through a more compound framework. By far, the most commonly used theories in the promotion of BCS are the Transtheoretical Model (TTM), the Theory of Planned Behavior (TPB), the Health Promotion Model (HPM), the Social Cognitive Theory (SCT), the Health Belief Model (HBM), and the Socio-Ecological Model (SEM).

Apart from BCS research, the effectiveness of health behavior theories has been proven to improve screening behaviors in other kinds of cancer. The Integrated Behavioral Model used by Serra et al. [29], for example, which involves constructs from the most commonly used theoretical models in health promotion (i.e., the Theory of Reasoned Action (TRA), the SCT, the HBM, and the TPB) was found to be successful to improve colorectal cancer screening. Another study by Huang et al. [30] suggested that variables pertinent to the TPB could successfully predict colorectal cancer screening uptake. Further, the HBM was effective to improve colorectal cancer screening uptake in general [31]. Cervical cancer screening, on the other hand, was predicted by using the TPB [32,33]. Additionally, Shida et al. [34] and Aldohaian et al. [35] found that the HBM and the TRA have a positive effect on cervical cancer screening outcomes. Apart from that, the HBM could positively affect prostate cancer-preventive behaviors by improving individual knowledge level and leaving positive effects on their health beliefs [36].

Previous reviews on the effectiveness of educational interventions revealed improved screening rates with culturally relevant components, multiple strategies, language-appropriate interventions, multilevel interventions, personal and cognitive interventions, and model-based educational interventions [37,38,39,40,41,42]. However, these studies only sampled participants from specific countries or regions, which do not offer an international view [39,40,42,43]. Moreover, previous reviews focused on MMG uptake [39], knowledge of BC and BCS, and model-based intervention studies [38], while beliefs have not been explored [21,41,43,44]. Furthermore, the instruments used to evaluate the outcomes affect the quality of findings. The use of standardized, valid, and reliable tools assures high-quality data and simplifies the interpretation of the findings. However, previous reviews found that little is known about the tools used to measure outcomes. A good understanding of these points can help in the development of an effective BCS program for women. Although the program content, methods of delivery, and the person who delivers the intervention play a critical role in improving the knowledge and behavior towards BCS among women, these features have only been reviewed in brief. In this systematic review, these features are reviewed in further detail.

Overall, this systematic review primarily aims to evaluate the effectiveness of educational interventions on BCS uptake, knowledge, and beliefs among women in different parts of the world. A comprehensive assessment of these interventions potentially offers evidence on their efficacy for the development of future projects to address BC. This systematic review also presents valuable recommendations on the content and methods of delivery of such programs based on current evidence. Such programs are expected to promote BCS uptake and subsequently reduce the morbidity and mortality rates of BC in the long run.

## 2. Materials and Methods

The review was registered and conducted according to the PRISMA guidelines (Supplementary Table S1) with the registration number: CRD42020148423.

### 2.1. Literature Search

**Figure 1:**
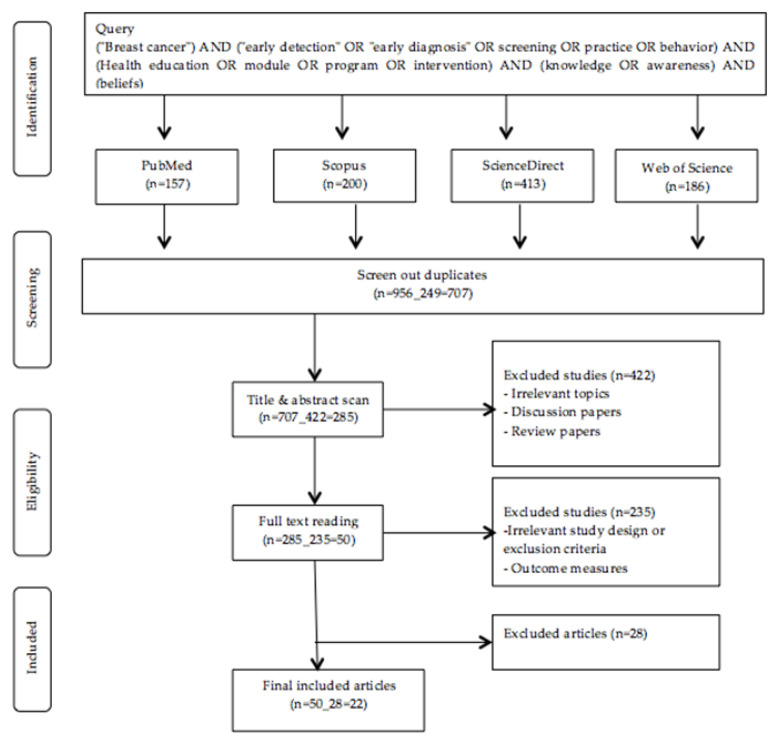
Flow chart of study selection.

For this review, a comprehensive literature search on four electronic databases, specifically PubMed, Scopus, Web of Science, and ScienceDirect, was conducted in May 2019. In order to identify relevant articles, a combination of keywords was used (Figure 1). The inclusion and exclusion criteria were used to evaluate the gathered articles (Table 1). These criteria were executed in Figure 1. Two reviewers independently extracted data from the studies. Search strategy can be seen in the Supplementary Table S2.

The Effective Public Health Practice Project (EPHPP) tool for intervention design studies was used to measure the quality of these studies. Using this tool, six domains, specifically research design, confounders, selection bias, blinding, data collection method, and dropouts and withdrawals, were evaluated. Each domain was valued as weak (one point), moderate (two points), or strong (three points). As for the total score, domain scores were averaged. As shown in Table 2, the quality of the studies was rated according to the following range of values: (1) weak quality: values of between 1.00 and 1.50; (2) moderate quality: values of between 1.51 and 2.50; (3) strong quality: values of between 2.51 and 3.00 [45,46]. Two reviewers independently evaluated the rating of these studies. Any arising disagreement on the rating was resolved with the involvement of a third reviewer.

### 2.2. Data Synthesis

The characteristics and results of the studies are summarized in both narrative and tabular forms can be found in the Table 2.

## 3. Results

The literature search on four electronic databases yielded a total of 957 articles. After reviewing the titles and abstracts, 422 articles were removed, resulting in a total of 50 full-text articles for further review. Following that, 28 articles were excluded after the application of the inclusion criteria. Hence, a total of 22 articles were retained (Figure 1). 

### 3.1. Characteristics of Study

#### 3.1.1. Participants and Setting

As shown in Table 2, the gathered studies were published between 2014 and 2019, and were conducted in Malaysia (n = 1) [16], Iran (n = 7) [19,47,48,49,50,51,52], United States (n = 6) [53,54,55,56,57,58], Turkey (n = 4) [10,11,59,60], Israel (n = 1) [9], United Arab Emirates (n = 1) [61], China (n = 1) [62], and India (n = 1) [63]. In addition, the participants were aged 18 years and above. With sample sizes of between 38 and 598, the participants were enrolled from communities (n = 12) [9,10,11,53,54,55,56,57,58,59,60,61] and healthcare settings (n = 6).

#### 3.1.2. Conceptual Framework

As illustrated in Table 2, the conceptual framework was explained in 18 studies. Multiple models were used as a guide in the intervention development of five studies, where Fathollahi-Dehkordi and Farajzadegan [48], Lee-Lin et al. in 2015b [53], and Lee-Lin et al. in 2015a [57] employed the HBM and TTM, while Taymoori et al. [52] employed the HBM and The TPB and Tuzcu et al. [10] employed the HBM and the HPM.

The HBM was the most commonly used model (n = 10) [11,16,19,47,49,50,51,56,60,62]. The HBM theoretical framework incorporates six constructs, namely: (1) perceived susceptibility, (2) perceived severity, (3) perceived benefits, (4) perceived barriers, (5) cues to action, and (6) self-efficacy. This model emphasizes that threats from health problems can affect health behavior. Women who perceive susceptibility to BC risk or believe that the disease is a serious problem are more likely to do the screening test. Besides this, women who experience more benefits and fewer barriers to the screening test, have high health motivation, and can successfully perform a behavior are also more likely to do the screening test [64,65]. Diverse demographic factors and knowledge may also influence their perceptions and indirectly impact their health-related behaviors, as suggested by the model [66].

Meanwhile, the TTM framework explains health behavioral change as a continuum of stages. One would advance from pre-contemplation (the stage of not thinking about the behavioral change) to the stage of contemplation (thinking about the change) before advancing to the action stage (implement the behavior) and finally, maintaining that behavioral change [67]. On the other hand, according to TPB, one’s behavioral achievements are influenced by motivation (intention) and ability (behavioral control). This theory distinguishes three forms of beliefs, specifically behavioral belief, normative belief, and control belief [66]. Focusing on three points, specifically one’s features and experiences, behavior-specific cognition and affect, and behavioral outcomes [68], the HPM views health as a positive dynamic condition, rather than the absence of disease. The increase in the level of one’s well-being is directed by health promotion. The model explains the multidimensional nature of individuals, as they react within the environment to maintain health.

Apart from the above theories, Gondek et al. [58] and Goel and O’Conor [55] applied the SCT (n = 2) whereas Elder et al. [54] applied the EM (n = 1). The SCT explains that one’s health behaviors are influenced by personal experiences, environmental factors, and actions of others. In order to achieve a particular behavioral change, the theory provides ways for social support to achieve behavioral change by instilling expectations and self-efficacy and utilizing observational education and any other reinforcements [69]. Meanwhile, as for EM, multiple levels of effect are considered to explore the behavioral change that guides the evolution of further comprehensive interventions [70].

#### 3.1.3. Intervention Strategies

As shown in Table 2 and Table 3, studies assigned the participants either into intervention or control groups individually (n = 13) [9,10,16,47,48,49,50,52,53,55,56,57,60] or through a unit-based approach (n = 3) [19. 51, 54]. The theory- and language-based approaches were the common characteristics of these educational interventions. With multiple intervention strategies, these programs were mostly performed in community and healthcare settings. All studies reported the utilization of linguistically appropriate methods in delivering interventions, of either spoken and written materials (n = 16) [9,10,16,19,47,49,51,52,53,54,56,57,59,60,61,62] or only spoken materials (n = 6) [11,48,50,55,58,63]. Furthermore, the program content in most of the studies mainly covered the key messages on normal breast anatomy, knowledge of BC and screening methods, knowledge on the significance and usefulness of screening methods, and health beliefs about BC and BCS. Table 3 shows intervention characteristics and findings in more detail.

The intervention methods of delivery varied across studies, where 19 studies reported multiple strategies in delivering the interventions, such as PowerPoint presentation (PPT), group discussion, video demonstration, training, relevant images, cards, and brochures [9,10,16,19,47,48,49,50,51,52,53,54,56,57,58,59,60,61,62]. Three studies delivered the interventions by a single strategy, either by PPT [11,63] or video demonstration [55].

Besides that, a total of 13 studies reported that the researcher(s) or main investigator(s) personally delivered the interventions [10,11,16,35,47,49,50,52,56,57,59,60,62]. Meanwhile, Gondek et al. [58] reported that a project director and/or health educator led the program delivery whereas Goel and O’Conor [55] reported that healthcare providers delivered the intervention. On the other hand, community healthcare workers provided the intervention in Elder et al. [54], while Mirmoammadi et al. [51] reported that a consultant delivered the intervention. In another study, Fathollahi-Dehkordi and Farajzadegan reported that a peer educator delivered the intervention [48]. However, four studies did not specify who delivered the intervention [9,19,61,63].

Other relevant strategies were also incorporated to handle the participants’ needs, which included the involvement of peer support (n = 1) [52], BC survivors (n = 2) [52,58], spouses (n = 1) [60], and female physicians (n = 1) [58] in the intervention. Furthermore, a personally and culturally tailored delivery was employed in four studies [9,53,57,60] and individual consultation was employed in five studies [10,51,53,56,57].

## 4. Outcome Measures and Study Results

Most of these studies assessed the effectiveness of the programs by measuring all three outcomes (i.e., BCS uptake, knowledge of BC, and beliefs about BC) (n = 14). Eight studies assessed the effectiveness of the programs by measuring only one or two outcomes from: (1) screening uptake (n = 1) [9]; (2) knowledge (n = 2) [61,63]; (3) knowledge and screening uptake (n = 1) [58]; (4) knowledge and beliefs (n = 1) [53]; (5) beliefs and screening uptake (n = 3) [10,52,57]. The common variables reported at baseline were age (in all studies), marital status (in all studies, except four studies [53,55,58,59]), educational level (in all studies, except one study [53]), and income (in 11 studies [9,10,16,48,52,54,55,56,57,59,62]). The family history of BC was reported in 10 studies [9,16,48,49,50,52,57,59,61,62]. Only one study did not specify any baseline variables [63]. Focusing on outcome measures, Table 3 displays the effects of the interventions, and Table 4 displays the details of the instruments.

### 4.1. Breast Cancer Screening Uptake

A total of 18 studies employed different methods to assess the participants’ BCS uptake and revealed conflicting results. Apart from the self-reporting found in six studies [16,49,52,55,56,57], Taymoori et al. [52] and Goel and O’Conor [55] used medical reports and review charts. On the other hand, 12 other studies used different questionnaires to evaluate the participants’ BCS uptake [9,10,19,47,48,50,51,54,57,58,59,60,62].

Meanwhile, seven studies that explored the practice of BSE among women reported consistent results. There were five experimental studies that involved two groups [9,10,16,19,50] and revealed higher BSE among the participants in the intervention group, as compared to the participants in the control group (*p* < 0.05), after the intervention. Likewise, two pre–post studies found a significant improvement in the rate of BSE performance among the participants after the intervention [59,62]. However, six studies on the CBE uptake reported conflicting results. Freund et al. [9] and Masoudiyekta et al. [19] did not find any significant difference between the two groups after the interventions (*p* > 0.05), while four other studies showed that the intervention groups recorded significant levels of CBE performance, as compared to the control groups (*p* < 0.05) [10,48,51,54].

Additionally, a total of 13 studies assessed the changes in MMG uptake among the participants after they received education but reported contradictory results. Five studies revealed that the MMG uptake improved significantly among women in the intervention group after they received education, as compared to those in the control group (*p* < 0.05) [10,19,51,54,55]. In another study, Taymoori et al. [52] found that, after adjusting for marital status and healthcare centers, the participants of HBM (AOR = 5.11, CI = 2.26–11.52, *p* < 0.001) and TPB (AOR = 6.58, CI = 2.80–15.47, *p* < 0.001) groups were five and six times more likely, respectively, to obtain MMG relative to the participants of control groups. Likewise, Lee-Lin et al. in 2015a [57] revealed that, after controlling for age, marital status, and age when participants moved to the United States, women in the intervention group were nine times more likely to complete MMG than women in the control group (AOR = 9.10, CI = 3.50–23.62, *p* < 0.001). Wu and Lin [56] further implemented a sub-group analysis of age, length of residence in the United States, and insurance status to evaluate the intervention’s effect on MMG uptake. A significant difference between groups was observed in participants with insurance coverage for MMG (56% in the intervention group versus 34% in the control group) (*p* = 0.03) and elderly women (65 years or older) (51% in the intervention group versus 25% in the control group).

When it comes to comparing different educational interventions, Heydari and Noroozi [49] showed that, after the intervention, 80% of the participants in group training and 33% of the participants in the multimedia group practiced MMG (*p* = 0.003). Meanwhile, Seven et al. [60] did not find any statistically significant difference in the screening rate among the three methods of education (*p* = 0.067). However, women who were involved in a group had a higher rate of MMG screening than women who were educated individually (*p* = 0.034).

As for the ultra-Orthodox Jewish group, Freund et al. [9] found a significantly greater number of women from the intervention group performed MMG screening (*p* = 0.009) after the intervention, as compared to the non-intervention group. On the other hand, when it came to the Arab population group, there was no significant difference between the intervention and control groups (*p* > 0.05). Apart from that, Gondek et al. [58] and Kocaöz et al. [59] employed a pre–post study design and reported that 33.0% and 28.4% of women in the respective post-intervention assessment completed MMG. The scores of BC behaviors were found to significantly improve among women in the intervention group, as compared to women in the control group, after the intervention (*p <* 0.05) [47].

### 4.2. Knowledge of Breast Cancer and Breast Cancer Screening

A total of 16 studies reported contradictory results on the changes in BC and BCS knowledge levels among the participants (before and after intervention). Different tools were used to evaluate the knowledge levels, where higher scores indicate greater knowledge. There were five studies that used valid and reliable knowledge tests [16,47,50,61,63]. Three studies reported that the participants in the intervention group recorded significantly higher knowledge scores than those in the control group [16,47,50]. Likewise, Vasishta et al. [63] and Rabbani et al. [61] employed pre–post study designs and observed a significant increase in BC knowledge (between the pre- and post-intervention).

Meanwhile, six studies reported on either the validity or reliability of the knowledge tests [19,49,51,53,60,62]. Although the knowledge scores of both intervention and control groups in the post-test in a study by Heydari and Noroozi [49] showed a significant increase (*p* < 0.001), no significant difference in the knowledge scores between both groups was reported (*p* = 0.128). Likewise, Seven et al. [60] reported a statistically significant improvement in the knowledge mean scores after intervention for each group of the three methods of education (*p* < 0.001) but no significant difference in the knowledge mean scores among the three groups (*p* > 0.05). Three studies showed that, after the intervention, the knowledge scores of the intervention group were significantly higher than that of the control group [19,51,53]. In addition, the participants’ knowledge scores in a study by Ouyang and Hu [62] increased significantly after the intervention (*p* < 0.013).

Besides that, five more studies assessed changes in the knowledge level but the psychometric properties of the instruments used in these studies were not reported [11,48,54,55,58]. Fathollahi-Dehkordi and Farajzadegan [48] showed a significant mean difference in the knowledge scores between the control and intervention groups following the intervention (*p* < 0.001). However, Goel and O’Conor [55] and Elder et al. [54] reported no significant differences in the knowledge scores of both groups after education (*p* > 0.05). Nevertheless, Goel and O’Conor [55] reported a significantly higher knowledge score among the participants in the intervention group (*p* = 0.04). Gondek et al. [58] and Yılmaz et al. [11] employed the pre–post study design and detected a significant improvement in the knowledge mean scores from the pre-test assessment to the post-test assessment (*p* < 0.05).

### 4.3. Health beliefs of Breast Cancer and Breast Cancer Screening

Inconsistent results were reported on modifications in the beliefs about BC and BCS (n = 15). A total of 14 studies applied the standard validated Champion’s HBM scale in different languages [10,11,16,19,47,48,49,50,51,52,53,59,60,62] using only certain model subscales or all model subscales to assess the changes in the health beliefs about BC and BCS, which are as follows: (1) perceived susceptibility, perceived benefits, and perceived barriers [53]; (2) perceived susceptibility, perceived severity, perceived benefits, perceived barriers, and health motivation [48,49,60]; (3) perceived susceptibility, perceived severity, perceived benefits, perceived barriers, and self-efficacy [19,47,50,52,62]; (4) perceived susceptibility, perceived severity, perceived benefits, perceived barriers, health motivation, and self-efficacy [10,11,16,51,59].

For instance, Lee-Lin et al. in 2015b [53] found that women in the education group recorded higher perceived susceptibility (*p* < 0.01) and lower perceived barriers to BC (*p* < 0.05) in the post-intervention than the control group. In another study, Heydari and Noroozi [49] found a significant decrease in the perceived barriers for both groups following the intervention (*p* < 0.05). As for the education group, health motivation (*p* = 0.01) and perceived benefits of MMG (*p* = 0.003) were found higher in the post-test but no statistically significant differences were reported in the multimedia group regarding perceived susceptibility, perceived severity, perceived benefits of MMG, and health motivation (*p* > 0.05). Conversely, the comparison of both education and multimedia groups showed no changes in the perceived barriers, perceived susceptibility, and perceived severity subscales (*p* > 0.05) but health motivation (*p* = 0.04) and perceived benefits (*p* = 0.029) were found higher in the education group as compared to the multimedia group.

Adding to that, Fathollahi-Dehkordi and Farajzadegan [48] found that all constructs of health beliefs were significantly impacted by time and time–group interaction (*p* < 0.001). The effect of the group factor was found to be significantly associated with perceived sensitivity, perceived benefits, and health motivation subscales (*p* < 0.05). Meanwhile, Seven et al. [60] reported no significant differences among the three educational groups in the scores of all subscales of health beliefs before and after education (*p* > 0.05), except for the scores of the health motivation subscale. Eskandari-Torbaghan et al. [47] found that, after the intervention, perceived susceptibility, perceived benefits, and perceived barriers improved significantly among women in the intervention group, as compared to women in the control group (*p <* 0.05). However, no statistically significant differences between both groups for the subscales of perceived seriousness and self-efficacy (*p* > 0.05) were reported.

Two studies revealed an increase in the mean scores for all HBM constructs for the experimental group, as compared to the control group, after the intervention (*p* < 0.05) [19,50]. Ouyang and Hu [62] employed a pre–post study design and found significant improvement in the perceived benefits, confidence, and perceived seriousness among the participants after the intervention (*p* < 0.05). However, no statistically significant differences for perceived susceptibility and perceived barriers (*p* > 0.05) were observed. The participants demonstrated substantial changes in all HBM and TPB constructs (*p* < 0.05) [52].

On the other hand, Akhtari-Zavare et al. [16] reported that the intervention group recorded significant changes in the scores of perceived benefits of BSE (*p* < 0.001), perceived barriers of BSE (*p* < 0.01), the confidence of doing BSE (*p* < 0.001), and total health beliefs (*p* = 0.04) after education compared to the control group. However, the study found no significant differences between the intervention and control groups for the remaining components (*p* > 0.05). On the other hand, Kocaöz et al. [59] reported significant improvements in health motivation (*p* = 0.03), perceived barriers of BSE (*p* = 0.007), confidence of doing BSE (*p* < 0.0001), perceived benefits of MMG (*p* = 0.008), and perceived barriers of MMG (*p* = 0.001) after the intervention. No significant improvement in perceived susceptibility, perceived seriousness, and perceived benefits of BSE (*p* > 0.05) was found.

In another study, Mirmoammadi et al. [51] found that the post-intervention assessment detected significant changes in the HBM constructs (*p* < 0.05) between the intervention and control groups, except for perceived susceptibility and perceived severity (*p* > 0.05). Tuzcu et al. [10] also found higher perceived susceptibility (*p* = 0.04), health motivation (*p* < 0.001), perceived benefits of BSE (*p* < 0.001), and self-efficacy (*p* < 0.001) but lower perceived barriers of BSE and MMG (*p* < 0.001) in the intervention group compared to the control group after education. The study also reported no significant differences in perceived seriousness (*p* = 0.400) and perceived benefits of MMG (*p* = 0.137) after education.

Furthermore, adopting a pre–post study design, Yılmaz et al. [11] found that the mean scores of all HBM subscales improved significantly (*p* < 0.05). Using the 1990 Tampa survey items [54], the cancer screening group recorded significantly lower scores in perceived barriers, as compared to the physical activity group (*p* = 0.008).

## 5. Discussion

The continuous increase of BC among women has prompted researchers to propose different educational interventions to improve knowledge and beliefs about BC and BCS and subsequently, promote BCS uptake among women. The current systematic review focused on published intervention studies to assess the effects of these programs on BCS uptake, knowledge, and beliefs and to provide information on the characteristics of these interventions.

A standardized and validated instrument, namely the HBM scale developed by Champion [64] and Champion [65], was found to be commonly used by studies to assess health beliefs. However, different instruments were used by these studies to measure the knowledge, beliefs, and screening uptake of BC. Although self-reporting is an easier means to evaluate the BCS uptake, over-reporting may occur. Women may give socially desirable responses, rather than revealing their actual practice [42]. A medical report is believed to be a reliable method in terms of data accuracy to verify the BCS uptake accuracy. However, retrieving these records can be rather difficult.

Meanwhile, a linguistically convenient approach is commonly adopted as an effective intervention strategy to promote understanding among women. Multiple health models and theories (HBM, EM, HPM, SCT, TTM, and TPB), which were adopted in most of these studies, revealed health beliefs, knowledge of BC, and screening as mediators as well as the final target screening behavior. Hence, no positive behavior may take place without addressing and observing these mediators properly during the intervention design. In this systematic review, secondary outcome variables (knowledge and health beliefs about BC and BCS) were included since one or a combination of these variables can potentially affect the screening uptake change. Most of these studies found that HBM-based educational interventions successfully improved screening rates. Similar findings were reported in another review based in Turkey [40]. The current review is consistent with the previous review that explored the efficiency of theory-based interventions in elevating the BCS uptake [38].

Furthermore, most of the studies revealed favorable outcomes after the educational intervention, which were in line with another review by Agide et al. [21]. The reviewed studies used either single or multiple health behavior models or theories to improve BCS uptake, where the approach of multiple models appears to be more efficient in meeting the multidimensional needs of women [10,48,52,57]. These findings are consistent with the previous systematic reviews conducted by Naz et al. [38] and Secginli et al. [40]. Furthermore, the intervention content plays an essential role in achieving the target outcome. The current review provides detailed information on intervention content, which is consistent with previous work by Chan and So [42].

Suitable instrument selection to measure outcomes can assist in producing high-quality data. The adoption of the same instrument can help to facilitate comparisons of these studies. The variation in the instrument content may create difficulties in comparing these studies. Moreover, certain studies did not report the reliability and/or validity of their instruments, which makes the quality of their data questionable. Thus, the interpretation of the findings must be taken with caution.

Nonetheless, the strength of this systematic review is the inclusion of experimental studies that were conducted in high- and middle-income countries. However, several limitations of this systematic review are also identified. Firstly, only published articles from 2014 to 2019 were gathered for the review. Secondly, the variation in the study designs and methods utilized in the included studies made pooling of results impossible. Thirdly, the variation in the follow-up length in these studies also limited the ability to compare the efficiency of interventions across studies. Apart from the varying validity and reliability of instruments used, the use of different instruments to assess outcomes was also another limitation.

## 6. Conclusions

Despite the observed improvements in women’s knowledge, beliefs, and screening practices following educational interventions, the current systematic review revealed a difficulty in proving the effectiveness of such programs given the inconsistencies in the reported findings. These discrepancies imply the need for more research. The implementation of a future comprehensive program that links the most effective intervention characteristics, mediators, and factors that influence BCS outcomes is suggested. Additionally, the discussed findings of this review present significant implications for researchers, healthcare workers, and intervention planners to produce BCS health educational interventions that target women. The knowledge obtained from this review can be used to design comprehensive BCS programs, which can help to strengthen the existing healthcare systems with the purpose of disseminating proper knowledge to wider communities.

## 7. Recommendations

As a result of the current systematic review, we present several significant recommendations on the development of educational interventions for higher BCS uptake. Firstly, it is recommended for future research to comprehensively examine educational interventions in order to provide strong evidence on their effectiveness. Additionally, to produce high-quality data, more reliable resources to evaluate screening practices are recommended, such as medical reports and the use of standardized validated instruments, such as the CHBM scales to assess health beliefs, rather than relying on questionnaires.

Furthermore, we recommend the implementation of theoretically based interventions to promote women’s BCS behaviors. Theoretical and model-based programs are more successful than programs that are not based on theories or models since these programs are based on an accurate understanding of the health behavior mechanism changes which result in successful plans. Moreover, we suggest that the multiple model based educational intervention approach is to be utilized as guidance for interventions to understand the cultural and psychosocial factors that influence BCS behaviors. Educational interventions that employ a multiple models method have been found to be more effective in meeting women’s’ multidimensional needs [52]. Such models account for social, cultural, and economic barriers which may promote women’s health beliefs.

Besides that, the intervention content should include key messages on knowledge and beliefs related to BC and BCS, and information on the importance and effectiveness of screening tests. Moreover, the intervention programs with the use of live demonstration (such as videos and printed materials) can be implemented individually or with in-group sessions by healthcare providers or lay instructors. Powerful and well-designed RCTs that provide a detailed description of the study and intervention are necessary.

## Figures and Tables

**Table 1:** Inclusion and exclusion criteria.

Types of Studies to Be Included	Types of Studies to Be Excluded
- Randomized control trials (RCTs) or pre–post studies. - Articles that provided educational interventions, health promotions, and behavioral interventions designed to improve BCS (breast cancer screening) knowledge and/or beliefs and/or BCS uptake.	- Abstracts. - Focused on BC (breast cancer) survivors, individuals with BC, or treatment and rehabilitation. - Non-intervention studies, irrelevant study designs (e.g., descriptive research), and studies that did not report quantitative valid outcome measures (e.g., studies that did not report values such as, frequency, percentage, mean (SD), P-value, or confidence interval).
Participants Women aged eighteen years old and above, without history of BC.	
Intervention Health promotion and behavioral program on BCS targeted women.	
Control group Either usual care or no intervention, or minimal intervention or intervention other than the intervention groups.	
Outcomes Any of the following: BCS uptake (BSE (breast self-examination), CBE (clinical breast examination), MMG (mammography)), knowledge of BC and screening, health beliefs about BC and screening.	
Publication year From January 2014 to May 2019.	
Language English.	
Article type Original studies.	

**Table 2:** Study characteristics.

Study/Country	Study Design/EPHPP	Theory	Participants Characteristics/Sample size	Setting/Unit of Assignment
- Akhtari Zavare et al. 2016 [16] - Malaysia	- RCT - Strong	- HBM	- Undergraduate students - 20 years old and above - N = 370 (I:186; C:184)	- 2 selected public universities - Individual-based
- Heydari and Noroozi, 2015 [49] - Iran	- RCT - Moderate	- HBM	- Female elementary school teachers - Older than 40 years - N = 120 (G1:60; G2:60)	- 21 elementary schools - Individual-based
- Eskandari-Torbaghan et al. 2014 [47] - Iran	- Controlled clinical trial - Moderate	- HBM	- Female staff - Age for intervention: (M = 35.38, SD = 8.01), Age for control: (M = 34.39, SD = 8.98) - N = 130 (I:65: C:65)	- University - Individual-based
- Goel and O’Conor, 2016 [55] - USA	- Quasi-experimental - study - Moderate	- SCT	- Women with low levels of formal education - 40 years old or older - N = 97 (I:49; C:48)	- Community health center - Individual-based
- Kocaöz et al. 2017 [59] - Turkey	- Semi experimental (in a - single group) - Moderate	- No theory	- Women residents in villages and towns - 18 years old and above - N = 342	- Mosques, schools, and cafés
- Elder et al. 2017 [54] - USA	- RCT - Strong	- EM	- Self-identified Latina - 18–65 years old - N = 436 (I:219; C:217)	- Churches - Church unite
- Yılmaz et al. 2017 [11] - Turkey	- Semi-empirical pre - post-test - Moderate	- HBM	- Women aged 20 years and above - N = 244	- Community education center
- Freund et al. 2017 [9] - Israel	- RCT - Moderate	- No theory	- Arab and Ultra-Orthodox Jewish women - 40–60 years old - N = 598, Arab N = 331 (I:24; C:90), Jewish N = 267 (I:148; C:119)	- Faith-based communities in Israel - Individual-based
- -Mirmoammadi et al. 2018 [51] - Iran	- RCT - Moderate	- HBM	- Women attending healthcare centers - Aged > 40 years - N = 140 (I:75; C:75)	- Healthcare centers - Health center-based
- Khiyali et al. 2017 [50] - Iran	- Quasi experimental - study - Moderate	- HBM	- Women attending health centers - Older than 20 years - N = 92 (I:46; C:46)	- Two health centers - Individual-based
- Masoudiyekta et al. 2018 [19] - Iran	- Quasi experimental - study - Moderate	- HBM	- Women referred to health centers - 20–60 years old - N = 226	- Health centers - Health center unit
- Lee-Lin et al. 2015b [53] - USA	- RCT - Moderate	- HBM and TTM	- Chinese American immigrant women - 40–85 years old - N = 300 (I:147; C:153)	- Community organizations - Individual-based
- Lee-Lin et al. 2015a [57] - USA	- RCT - Moderate	- HBM and TTM	- Chinese American immigrant women - 40–85 years old - N = 300 (I:147; C:153)	- Community organizations - Individual-based
- Fathollahi-Dehkordi and Farajzadegan, 2018 [48] - Iran	- RCT - Moderate	- HBM and TTM	- Women referring to a hospital who have 1st or 2nd degree family history of BC - 20 years old or older - N = 98 (I:48; C:50)	- Hospital - Individual-based
- Taymoori et al. 2015 [52] - Iran	- RCT - Moderate	- HBM and TPB	- Iranian women - 50 years or older - N = 184 (intervention based on HBM + TPB: 60; intervention based on the HBM: 63; C:61)	- 3 healthcare centers - Individual-based
- Rabbani et al. 2019 [61] - United Arab Emirates	- One-group pre post-test - experimental design - Moderate	- No theory	- Arab women - 18–65 years old - N = 250	- Community
- Gondek et al. 2015 [58] - USA	- One-group pre post-test - design - Moderate	- SCT	- Immigrant and refugee females - N = 348	- Resettlement agency locations
- Ouyang and Hu, 2014 [62] - China	- One-group pre post-test - design - Moderate	- HBM	- Female residents - Over 20 years old - N = 38	- Community health center
- Seven et al. 2014 [60] - Turkey	- Interventional study de - signed - Moderate	- HBM	- Female aged 50–69 years old - N = 327 women (individual education group: 115; individual education plus an educational brochure for the spouse group: 112; group education: 100)	- Participants’ houses - Individual-based
- Tuzcu et al. 2016 [10] - Turkey	- Quasi-experimental - study - Moderate	- HBM and HPM	- Migrant women - Older than 20 years - N = 200 (I:100; C:100)	- Family health center - Individual-based
- Vasishta et al. 2018 [63] - India	- One group pre post-test - design - Moderate	- No theory	- Female students - 18–25 years old - N = 177	- College
- Wu and Lin, 2016 [56] - USA	- RCT - Moderate	- HBM	- Chinese American women - 41 years or older - N = 193 (I:96; C:97)	- Chinese organizations and community centers - Individual-based

RCT, Randomized control trials; TTM, The Transtheoretical Model; TPB, the Theory of Planned Behavior; HPM, the Health Promotion Model; SCT, the Social Cognitive Theory; HBM, the Health Belief Model; EM, the Ecological Model.

**Table 3:** Intervention characteristics and findings.

Study	Intervention Characteristics	Findings
Akhtari Zavare et al., 2016 [16]	Content: Breast health awareness/normal breast/BC knowledge/screening methods/training on BSE performance. Method of delivery: Spoken/written materials. 2 h workshops with a group of 12–13, delivered by the study researchers (1 h lecture in the form of PPT + 1 h training on BSE and participant’s duplication on breast silicon model) + educational booklet. Control group: Received all materials after the completion of the education.	- Both groups differed significantly in their performance and frequency of BSE at 12 months (*p* = 0.0001). - A significant improvement in knowledge (*p* = 0.003) and BSE (*p* = 0.001) for the intervention than the control group. - There were significant changes from baseline to six and twelve months in BSE benefits (*p* < 0.001), BSE barriers (*p* = 0.01), confidence (*p* < 0.001), and total HBM scores (*p* = 0.04) in the intervention group compared to the control group.
Heydari and Noroozi, 2015 [49]	Content: Breast anatomy/warning against BC/perceived susceptibility and severity/benefit and barriers of MMG/MMG performance. Method of delivery: Spoken/written materials. Delivered by the study researchers. Group education: Two training sessions lasting 45–60 min with a 1-week interval. In the form of group discussion/oral presentation/PPT slides/SMS reminder. Multimedia education group: Training through CD/educational SMS/SMS reminder.	- A total of 80% in the group training and 55% in the multimedia group performed MMG (*p* = 0.003). - Significant improvement in knowledge scores in the two groups at the post-test (*p* < 0.001). However, a comparison between the two groups found no difference (*p* = 0.128). - In multimedia group: barrier showed a significant decrease (*p* = 0.007), no significant changes in susceptibility, severity, MMG benefit and health motivation (*p* > 0.05). - In group education: significant improvement in health motivation (*p* = 0.01) and MMG benefits (*p* = 0.003), and significant decrease in barriers (*p* = 0.006). - Comparison of the two groups found no significant difference in the susceptibility, severity, and barriers (*p* > 0.05), but health motivation (*p* = 0.04) and benefit (*p* = 0.029) were higher for the group education than multimedia group.
Eskandari-Torbaghan et al., 2014 [47]	Content: BC symptoms/right time for MMG/preventive behaviors of BC including healthy diet and physical activity/perceived sensitivity and seriousness, barriers in performing BC preventive behaviors. Methods of delivery: Spoken/written materials. Three sessions, delivered by the main investigators, each about 1–1.5 h long in the form of lectures/questions and answers/PPT/videos/educational booklet. Control group: Received training after the education completing.	- Behavior (*p* = 0.045), knowledge (*p* < 0.001), susceptibility (*p* = 0.005), benefits (*p* < 0.001), and barriers (*p* = 0.004) all improved significantly in the intervention group compared to the control group. - No significant differences between the two groups in seriousness and self-efficacy (*p* > 0.05).
Goel and O’Conor, 2016 [55]	Content: Importance of MMG/experience of undergoing MMG/BC grows and spreads. Method of delivery: Spoken. Delivered by healthcare provider. A brief 30-s video meeting between the provider and the participants. Control: Usual care.	- MMG completion rate was higher in the group education than the control group, 33% vs. 13% (*p* = 0.02). - An improvement in knowledge in the group education than the control group (*p* = 0.04); however, in pre–post test knowledge change between the groups was not statistically significance (*p* = 0.08).
Kocaöz et al., 2017 [59]	Content: Breast anatomy/unusual changes in the breast/importance of BC/risk factors/early diagnosis/symptoms/treatment/BSE. Method of delivery: Spoken/written materials. Delivered by the study investigators, a session of 40 min in the form of visual presentation/participants’ performance of BSE/an education brochure on BC and its screening/participation in screening programs.	- After education 28.4% of participants performed MMG, 69.9% practiced BSE, and 29.3% performed BSE regularly. - Significant improvement in health motivation (*p* = 0.03), barriers of BSE (*p* = 0.007), self-efficacy (*p* < 0.001), MMG benefit (*p* = 0.008), and MMG barriers (*p* = 0.001) were found, but no significant improvement in the scales of susceptibility, seriousness, and BSE benefits were observed (*p* > 0.05).
Elder et al., 2017 [54]	Content: Breast anatomy/importance of BC prevention/risk factors and treatment/prevention steps/myths on BC/perceived barriers. Method of delivery: Spoken/written materials. A 6-week series of classes for each 90–120 min delivered by a trained bilingual/bicultural community healthcare worker. The intervention is a multilevel model that includes: Individual-level: BC screening classes/handouts to participants. Interpersonal level: Two motivational interviewing (MI) calls/reminder calls. Organizational level: Cancer screening sessions were announced in the church brochure/churches assigned spaces for education sessions. Environmental Level: Through training, promotors give information about the services and the local clinics, and completed Affordable Care Act workshops. Control: Physical activity intervention.	- At 12-month follow-up, MMG and CBE practices were higher in the cancer-screening group (from 44% to 61%, *p* = 0.0004 and 47% to 63% *p* = 0.003 respectively) than in the physical activity group. - No significant difference was found for knowledge between the two groups (*p* = 0.95). - Barriers were lower in the group of cancer screening - than the group of physical activity (*p* = 0.008).
Yılmaz et al., 2017 [11]	Content: Symptoms and risk factors of BC/screening approaches (BSE, CBE, MMG). Method of delivery: Spoken. A 60–90 min PPT delivered by the study researcher.	- All knowledge subscales increased from pre- to post-test (*p* < 0.001). - All HBM subscales improved from pre- to post-test (*p* < 0.05).
Freund et al., 2017 [9]	Content: General screening recommendations/BC facts/early detection procedures/screening barriers, cultural and religious beliefs. Method of delivery: Spoken/written materials. The education was designed to be tailored culturally and personally for each participant through interview/discussion. Control: No treatment.	- Ultra-Orthodox Jewish group: - Significant improvement in the performance of BSE (*p* = 0.004) and MMG (*p* = 0.009) among respondents in the intervention group than non-intervention group. - No significant differences were observed between the two groups on CBE attendance (*p* > 0.05). - Arab group: - Significant number of respondents in the intervention group performed BSE (*p* = 0.002) as compared to the non-intervention group. - No statistically significant difference was found on CBE and MMG between the two groups (*p* > 0.05). - Factors predicting adherence to screening: - Religious beliefs with lower level and a well understanding of the significant of the screening were significant predictors of performing BSE and CBE in both groups.
Mirmoammadi et al., 2018 [51]	Content: HBM constructs/breast anatomy/physiological changes in the breast/symptoms and signs of BC/methods of BCS/treatment of BC. Method of delivery: Spoken/written materials, in 4 weekly sessions, 90 min long, of BCS individual consulting in the form of Q&A/speech/slideshow/group discussion/practical training/oral and practical test/booklet. Control: Routine care. After the study completion, the training booklet was offered to them.	- Three months following the intervention: - MMG increased significantly from 26.7% to 49.3% in the experimental group compared to the control group (*p* < 0.001). - CBE increased significantly from 29.3% to 52% in the experimental group compared to the control group (*p* = 0.041). - Significant changes were detected between the two groups on knowledge (*p* < 0.001). - Significant changes were detected between the two groups on HBM constructs (*p* < 0.05). However, susceptibility (*p* = 0.18), severity (*p* = 0.9), and MMG barriers (*p* = 0.14) did not show significant differences.
Khiyali et al., 2017 [50]	Content: Risk factors/complications/screening methods including BSE/when and how to correctly perform BSE. Method of delivery: Spoken. The training program included 5 one-hour training sessions delivered by the study researcher through group discussion/video demonstration/training sessions. Control: No treatment.	- After the intervention there was an elevation in the BSE behavior (*p* < 0.001), knowledge (*p* < 0.001), and HBM subscales ((*p* < 0.05) in the experimental group compared to the control group (*p* < 0.001).
Masoudiyekta et al., 2018 [19]	Content: BC facts and figures/BC epidemiology/risk factors, symptoms of BC/importance of early detection/screening methods/guidelines for MMG/health motivation/susceptibility to BC/benefits, barriers, and self-efficacy/list of public hospitals that provide MMG. Method of delivery: Spoken/written materials. Four 90–120 min teaching sessions in the form of PPT/videos/performing BSE on the models/group discussion/Q&A session/pamphlets. Control: No treatment.	- Three months after the intervention: - BSE and MMG increased significantly in the intervention group compared to the control group (*p* < 0.001). - No significant changes in CBE (*p* = 0.66) between the two groups. - Significant improvement in knowledge (*p* < 0.001) and HBM subscales (*p* < 0.05) among respondents in the intervention group compared to the respondents in the control group.
Lee-Lin et al., 2015b [53]	Content for the intervention group: BC incidence/risk factors/process and benefits of MMG/cultural barriers. Content for the control group: Brochure consists of the following: do you think you are at risk of BC, what is MMG screening, who should get MMG, why and how should I get MMG, how can I pay, and where can I find information. It also stressed taking care of oneself. Method of delivery: Spoken/written materials. The TBHEP 1 h intervention included 2 parts, group teaching delivered by the study researcher followed by individual counseling by trained staff/the materials covered cultural, graphic like photos of both old and young Chinese women, Asian landscapes, and some dialogs between a Chinese grandmother, mother, and daughter/PPT/group discussions/Q&A sessions/face-to-face interactions. Control: A brochure emphasized caring for self. Reminder of follow-up survey with telephone calls in 3 months.	- Three months following the intervention: - The education group had higher susceptibility compared to the control group (*p* < 0.01). - 12 months following the intervention: - The education group had higher knowledge than the control group (*p* < 0.05). - The education group had fewer barriers than the control group (*p* < 0.05). - No significant difference on perceived benefits was reported between the two groups at all time points.
Lee-Lin et al., 2015a [57]	Content for the intervention group: BC incidence/risk factors/process and benefits of MMG/cultural barriers. Content for the control group: Brochure consists of the following: do you think you are at risk of BC, what is MMG screening, who should get MMG, why and how should I get MMG, how can I pay, and where can I find information. It also stressed taking care of oneself. Method of delivery: Spoken/written materials. The TBHEP 1 h intervention included 2 parts, group teaching delivered by the study researcher followed by individual counseling by trained staff/the materials covered culturally graphics like photos of both old and young Chinese women, the Asian landscapes, and some dialogs between a Chinese grandmother, mother, and daughter/PPT/group discussions/Q&A sessions/face-to-face interactions. Control: A brochure emphasized caring for self. Reminder of follow-up survey with telephone calls in 3 months.	- Post intervention: - Women in intervention group had completed a MMG compared to women in the control group (*p* < 0.0001). - Six months post-intervention: - The intervention group was 9 times more likely to perform MMG compared to the control group (OR = 9.10, 95% CI: 3.50–23.62, *p* < 0.001).
Fathollahi-Dehkordi and Farajzadegan, 2018 [48]	Content: BC risk factors, signs and symptoms/screening tests/benefits of early diagnosis/ways to improve sensitivity, and severity of BC, methods to increase motivation, and overcoming barriers. Method of delivery: Spoken. Three sessions, 2 h long, in 4 groups with 10 to 15 women in three weeks, delivered by a peer educator in the form of oral presentation/image presentation/group discussion/women shared knowledge and beliefs on BC and BCS. The educator talked about her experiences and beliefs/women were also encouraged to connect with each other after the completion of the education and share their new practices to help one another to overcome screening barriers. Control: Invited to contribute in training session at the completion of the follow-up and education was offered to them. A telephone number for consultations were also provided to them.	- Three months post-intervention: - CBE increased from 14.6% to 52.1% in intervention group vs. 10% to 18% in control group (*p* < 0.001). - Most women in the intervention group were in the action stage of CBE compared to women in the control group who were in the contemplation stage (*p* < 0.001). - One and three months post-intervention: - Significant difference in the knowledge between the two groups (*p* < 0.001). - Significant difference in HBM subscales between the two groups (*p* < 0.05). - Knowledge and HMB subscales were improved by time–group interaction and time factors (*p* < 0.001). - The effect of group factor was associated only with knowledge, sensitivity, benefits, and health motivation subscales (*p* < 0.05).
Taymoori et al., 2015 [52]	The HBM intervention: Content: Perceived threat, MMG benefits and barriers, and self-efficacy. Method of delivery: Spoken/written materials. Eight sessions in the form of slides, pamphlets, films, group discussions, and role modeling with BC survivors. Delivered by research staff in groups of 5–12 women. Individual sessions tailored to women’s specific needs. Each woman received eight 45–60 min group sessions, women were divided into groups based on their reported requirements. The HBM + TPB intervention:Content: In addition to HBM intervention content, participants received sessions focused on subjective norms and perceived behavioral control. Method of delivery: Spoken/written materials. In addition to HBM methods of delivery, participants received 4 sessions on subjective norms and perceived control. Regarding subjective norms, small groups were formed to encourage peer support and to raise exposure to positive interpersonal norms, and education about the importance of developing social networks. In individual counseling sessions, participants were also asked to provide information for 5 relatives to remind them about MMG. Relatives were invited to participate in a 60 min session. Regarding perceived control, participants were trained to resolve environmental challenges. Reminder messages on scheduling MMG appointments and telephone conversations on subjective norms were also used. Control group: Received pamphlets following the completion of the follow-up survey.	- A significant increase in MMG screening in the HBM group (AOR = 5.11, 95% CI: 2.26–11.52; *p* < 0.001) and TPB group (AOR = 6.58, 95% CI = 2.80–15.47, *p* < 0.001) relative to the control group. - Comparable MMG screening rate was found between HBM and HBM + TPB interventions (AOR = 1.24, 95% CI: 0.58–2.6; *p* = 0.58). - Greater improvement among participants in the HBM and TPB constructs. - Significant differences found between the control and HBM participants (*p* < 0.05), while no significant differences found between the control and TPB participants on perceived benefits, susceptibility, and seriousness (*p* > 0.05). - No significant differences in the TPB among the HBM and TPB participants.
Rabbani et al., 2019 [61]	Content: General information on BC/signs and symptoms of BC/BC epidemiology, risk factors, anatomy, importance of early detection/BSE/MMG/screening procedures/treatment options. Method of delivery: Spoken/written materials. A 45 min session in the form of slide show + handouts.	- Knowledge increased significantly from 12.9 ± 5.9 to 19.3 ± 1.9 after the intervention (*p* < 0.05). There was a significant increase in all BC knowledge domains from pre- to post-test (*p* < 0.05).
Gondek et al., 2015 [58]	Content: BC statistics/risk factors, signs, symptoms of BC/myths of BC/methods of BCS (BSE, CBE, MMG). Method of delivery: Spoken. A health educator and/or project director delivered educational sessions. A 60–90 min breast health education program, evidence-informed, and community-based culturally competent. Session delivered in multiple languages, in the form of presentations/interactive breast model session/local BC survivor speaks about her personal experiences/a female physician to answer participants questions/women aged 40 years or older who were not currently practicing BCS were contacted and proposed one-on-one navigation assistance in completing BCS.	- After the intervention - A total of 33% of women >40 years old completed MMG (*p* < 0.001). - Knowledge increased on the post-program assessments (*p* < 0.05).
Ouyang and Hu, 2014 [62]	Content: Prevalence, characteristics, risk factors, and signs of BC/early detection methods/healthy diet and exercise guidance/importance and benefit of BSE/technique of BSE. Method of delivery: Spoken/written materials. The intervention delivered by the study researcher consisted of 1 h (20 min educational session, 30 min BSE training, 10 min discussion), in the form of PPT presentation/pictures/BSE color images/BSE diagrams/videos/booklet and shower card/monthly telephone follow-up.	- Significant improvement in BSE practice after training (*p* < 0.001). - Knowledge increased 1 and 3 months after the education (*p* < 0.01). - Significant improvement in benefits (*p* < 0.005), competency (*p* < 0.001), and seriousness (*p* < 0.032) after training. - No significant change was found in the barriers and susceptibility (*p* > 0.05).
Seven et al., 2014 [60]	Content: Both brochures provided information on BC early signs and symptoms, risk factors/importance of BSE, CBE, MMG/current recommendations on BSE, CBE, MMG. Method of delivery: Spoken/written materials. The primary investigator delivered the intervention in the participants’ homes. Individual education: Each participant received a one-on-one education + educational brochure. Individual education and husband brochure Each woman received one-on-one training and two educational brochures; one for her and another for her husband. Group education Some 60–90-min-long educational sessions + educational brochure/women invited to participate in free BCS services.	- No significant changes in screening rates were found between the three methods of education (*p* = 0.067). However, participants in-group education had a higher significant rate of MMG screening than participants in other groups (*p* = 0.03). - Significant increase in knowledge in each group (*p* < 0.001). However, no significant difference in knowledge between the three groups (*p* = 0.548). - Changes in HBM subscale among all groups were not statistically significant (*p* > 0.05), except for health motivation.
Tuzcu et al., 2016 [10]	Content: Breast anatomy/incidence, mortality rate, risk factors of BC/changes in the breast/BSE, CBE, MMG/instructions on doing BSE/importance of screening methods/susceptibility/benefits, barriers, confidence/benefits, barriers of MMG. Method of delivery: Spoken/written materials. One hour, 10-week-long training in groups of 8–12 individuals delivered by study team members, in the form of 20 min PPT for visual images/15 min film about BSE/20 min BSE training on a breast model/10 min delivery and explanation of the reminder cards/telephone consultations/telephone calls reminders in the 3^rd^ month/invitation card for free MMG/screening behaviors cards. Control: Usual care. After the post-test, study team offered one-to-one education on BCS and reminder cards (BSE card, BCS approaches card) to the control group.	- Six months post-intervention: - The rates of BSE (*p* < 0.001), CBE (*p* < 0.001), and MMG (*p* = 0.011) were higher among respondents of the intervention group than respondents of the control group. - The intervention group had higher susceptibility (*p* = 0.04), health motivation (*p* < 0.001), BSE benefit (*p* < 0.001), self-efficacy (*p* < 0.001), and lower barrier of BSE and MMG (*p* < 0.001) than the control group. - No difference in seriousness (*p* = 0.400) and MMG benefits (*p* = 0.137) subscales between the two groups.
Vasishta et al., 2018 [63]	Content: Anatomy and physiology of the breast/risk factors for BC/steps and importance of BSE. Method of delivery: Spoken, through PPT.	- Before the intervention, 3.95% of the participants had good knowledge, which significantly increased to 59.89% after the intervention (*p* < 0.05).
Wu and Lin, 2016 [56]	Content for the intervention group: Knowledge of BC/risk factors/MMG screening guidelines/perceived barrier, benefits and self-efficacy. Content for the control group: MMG brochure on breast health. Method of delivery: Spoken/written materials. Individual telephone counseling delivered by research investigators, through 1 h telephone calls interview/application (computer program). Control: Brochure.	- Four months after the intervention: - The intervention group had more MMG screening than the control group 40% vs. 33%, however, the difference was not statistically significant (*p* > 0.05). - Women who are 65 years or older, 51% of them in the intervention group and 25% of them in the control group had perform MMG. - Women who had insurance coverage, 56% of them in the intervention group and 34% of them in the control group performed MMG (*p* = 0.03).

**Table 4:** Instrument details.

Study and Outcome Measures	Data Collection Periods	Method of Evaluation and Content of Instrument	Psychometric Properties
Akhtari Zavare et al., 2016 [16] - BSE frequency - Knowledge of BC and BSE - HBM scales	- Baseline - 6 months after the intervention - 12 months after the intervention	- SAQ (self-administrative questionnaire) dual-language (English and Malay). - BSE frequency: measured by questions on multiple answer choices (“once a month”, “occasionally”, “other” and “never”). - Knowledge data form: 35 items included general facts of BC, BC symptoms, risk factors, BSE, CBE, and MMG. With responses of true, false, I do not know. A correct answer scored 1 and a wrong or unsure answer scored 0. - HBM scale contains 40 questions on the seriousness, susceptibility, BSE barriers and benefits, confidence, and health motivation. Scores rated on a five-point Likert scale that ranged from “strongly disagree” to “strongly agree”.	- The study reported content validity index (CVI), face validity, test–retest reliability. Kappa value for BC general facts (0.70–0.80), risk factors (0.52–0.97), BC symptom (0.70–0.97), CBE and MMG (0.80–0.90), and BSE knowledge (0.70–0.87). - Intra-class correlation coefficient for: seriousness (0.89–0.96), susceptibility (0.79–0.86), benefit (0.85–0.98), barrier (0.70– 0.80), confidence (0.88–0.97), motivation (0.92–0.98). - Kappa value for practice of BSE (0.82) and frequency of BSE (0.85).
Heydari and Noroozi, 2015 [49] - Knowledge - HBM scales - MMG screening	- Baseline - 1 week after the intervention - 3 months after the intervention	- SAQ. - Items included questions about knowledge on BC, a correct answer scored 1 and a wrong answer scored 0. - 30 questions on susceptibility, severity, benefits, barriers, and health motivation. Items scored by a 5-point - Likert scale that ranged from “strongly disagree” to “strongly agree”. - One question on MMG screening, with yes/no answer. If the response is no, another question on intention to do MMG was asked.	- Kuder Richardson coefficient of knowledge = 0.78. - Cronbach’s alpha coefficients of HBM ranged from 0.72 to 0.89 (health motivation to susceptibility).
Eskandari-Torbaghan et al., 2014 [47] - BC behaviors - Awareness - HBM scales	- Baseline - 1 month after the intervention	- SAQ. - 5 items assessed BC behaviors, with responses answered as: always, sometimes, often, and never. Scores ranged from 3 to 0. - 16 questions measured awareness, with scores of 2 for right response, 1 for no comment response, and 0 for wrong response. - HBM assessed through 6 items on susceptibility, 5 items on seriousness, 5 items on benefits, 5 items on barriers, 5 items on self-efficacy, 6 items on cues to action. Scores were rated on a 5-point Likert scale that ranged from totally agree (5) to totally disagree (0). Scoring for the cues to action construct was rated in percentages.	Accepted items had content validity ratio larger than 0.62 and content validity indices larger 0.79. Cranach’s alpha = 0.76.
Goel and O’Conor, 2016 [55] - BC knowledge - MMG screening	- Baseline - 3 days post-test	- Telephone interview. - Knowledge was measured using 10 items, with “true, false” answers, in 5 domains: family history of BC, BC symptoms, physical test findings, MMG curability, and effectiveness. - MMG screening was assessed through self-report questions + chart reviews.	Psychometric properties were not reported.
Kocaoz et al., 2017 [59] - BSE practice - MMG practice - HBM scales	- Baseline - 6 months after the intervention	- SAQ. - 16 questions on participation in early screening programs, reasons if the exam had not been performed, and opinions on future involvement in such programs. - HBM was measured through 52 items on susceptibility, seriousness, BSE benefits and barriers, confidence, health motivation, MMG benefits and barriers. Items rated on a 5-point Likert scale that ranged from strongly disagree to strongly agree.	- First evaluation Cronbach’s alpha = 0.82–0.88. - Second evaluation Cronbach’s alpha = 0.79–0.88.
Elder et al., 2017 [54] - MMG - CBE - BC knowledge - Barriers to screening	- Baseline - 12 months after the intervention - 24 months after the intervention	- SAQ. - Cancer screening behaviors were assessed using the 2010 Behavioral Risk Factor Surveillance System Survey (BRFSS). If the respondent answered yes, they were asked how long it had been since their last screening was obtained. Answer comprised of 5 options. - Knowledge was assessed using the 6 items of the “Esperanza y Vida” cancer knowledge questionnaire. Scores were evaluated based on the percentage of correct responses. - Barriers were evaluated using 9 items of the 1990 Tampa survey. Answers rated on a 5-point Likert scale that ranged from “strongly disagree to strongly agree”, with higher scores imply greater perceived barriers to screening.	Psychometric properties were not reported.
Yilmaz et al., 2017 [11] - Knowledge of BC and BCS - HBM scales	- Baseline - 1 week after the intervention	- Face-to-face interviews. - Knowledge was measured using 18 items, with “true or false” responses. A correct response was scored 1 and incorrect response 0. - A 5-point Likert scale rated the HBM scales.	- HBM pre-test Cronbach’s Alpha = 0.74–0.88. - HBM post-test Cronbach’s Alpha = 0.76–092.
Freund et al., 2017 [9] - Adherence to screening (BSE, CBE, MMG)	- Baseline - 3 months after the intervention	A telephone questionnaire of 22 items that included questions on socio-demographic factors, questions on adherence to MMG, CBE, and BSE screening, and questions on cultural health beliefs.	- Content validity tested by 4 professional experts. - Construct validity was assessing by determining the correlations between barriers of HBM and cultural barriers of the current study. - Cultural and religious perceptions of Arab women were tested among 300 Arab women. - Cronbach’s alpha for religious beliefs and being cured = 0.86, fear of social losses = 0.72, accessibility barriers = 0.71, and exposure barriers = 0.61. - Factor analysis revealed 5 factors that explained 63.13% of the variance.
Mirmoammadi et al., 2018 [51] - MMG screening - CBE performance - Knowledge - HBM scales	- Baseline - 1 month after the intervention - 3 months after the intervention	- SAQ. - BCS included questions about history of doing CBE and MMG, how many times they had done it, questions about the sources of information, and the reasons for not doing BCS. - Knowledge (44 questions) using a previously standard questionnaire, with response options of (yes, no, I do not know); correct response rated 1 and wrong and I do not know rated 0. - HBM scales (63 questions). A 5-point Likert scale assessed the responses; agree scored 5 and disagree scored 1.	- Knowledge Cronbach’s alpha = 0.96. - HBM Cronbach’s alpha = 0.87.
Khiyali et al., 2017 [50] - BSE performance - Knowledge on BSE - HBM scales	- Baseline - 3 months after the intervention	- SAQ. - BSE was tested using 6 items, with 2 options (1 score for I do and 0 score for I do not know/do not answer) - 20 items on knowledge (1 score for correct answer and 0 score for wrong answer) - HBM was assessed using questions on susceptibility (8 items), severity (8 items), benefits (6 items), barriers (6 items), self-efficacy (8 items). All scales were rated based on a 5-point Likert scale that ranged from totally agree (5) to totally disagree (1).	- The validity of the questionnaire items was evaluated with CVI of higher than 0.15, and CVR of higher than 0.77. - Exploratory factor analysis revealed 6 factors. - Face validity was conducted among 40 women. - Content validity: was evaluated through the opinions of 12 specialists and experts. - Cronbach’s alpha = 0.86. - The reliability values for knowledge, susceptibility, severity, benefits, barriers, and self-efficacy were 0.85, 0.75, 0.80, 0.79, 0.82, and 0.77, respectively.
Masoudiyekta et al., 2018 [19] - BSE performance - CBE performance - MMG performance - Knowledge - HBM scales	- Baseline - 3 months after the intervention	- SAQ. - 3 questions on BCS. - 19 questions to measure the level of awareness, 1 point for the right response and 0 point for false response. - Questions on HBM included, benefits, susceptibility, barriers, severity, self-efficiency, and cues to action. Rating in a 5-point Likert scale, from strongly agree (5) to strongly disagree (1).	- The questionnaire was assessed for validity by professional experts. - Accepted items had content validity ratio larger than 0.62 and content validity indices larger than 0.79. - Cronbach’s alpha for susceptibility = 0.90, severity = 0.82, benefits = 0.85, barriers = 0.97, self-efficacy = 0.82, and cues to action = 0.94.
Lee-Lin et al., 2015b [53] - Knowledge - Perceived susceptibility - Perceived barriers to MMG	- Baseline - 3 months after the intervention - 6 months after the intervention	- A telephone questionnaire. - Knowledge assessed using 11 items, with answers of “increase risk, decrease risk, and not sure”. Scores range from 0 to 10). Results reported as percentages and frequency counts. - Susceptibility (3 items) on a Likert-scale (range from 3 to 13). - MMG barriers (21 items) on a Likert-type scale (range from 4 to 70).	- HBM Cronbach’s alphas ranged from 0.64 to 0.90. - HBM subscales items were evaluated with: (1) A review of the literature (2) Validity through content and cultural experts (3) Scales were pretested and critiqued among 10 Chinese American immigrants.
Fathollahi-Dehkordi and Farajzadegan, 2018 [48] - CBE screening - Knowledge - Beliefs	- Baseline - 3 months after the intervention	- A telephone questionnaire. - CBE stages were evaluated using Rakoweski classification stages: a) Precontemplation, b) Contemplation, c) Relapse, d) Action, e) Maintenance. - 12 items (wrong-right checklist) were used to assess knowledge. Right response was given 1 score and wrong response was given 0 score. - Beliefs were evaluated by 5 subscales of CHBMS construct including, sensitivity (3 items), severity (7 items), barriers (10 items), benefits (6 items), and health motivation (7 items), with a Likert scale that ranged from strongly disagree “1” to strongly agree “5”.	Cronbach’s alpha for sensitivity = 0.82, severity = 0.84, barriers = 0.73, benefits = 0.72, and health motivation = 0.77.
Taymoori et al., 2015 [52] - Changes in the HBM and TPB constructs - MMG screening	- Baseline - 6 months after the intervention	- SAQ translated to Farsi language. - The questionnaire included items on socio-demographic factors and 37 items for both constructs (HBM + TPB) related to susceptibility (3 items), severity (7 items), MMG benefits (6 items), MMG barriers (9 items), self-efficacy (10 items), subjective norms (1 item), and perceived control (1 item). - The HBM was evaluated on a 4-point scale ranging from strongly disagree “1” to strongly agree “4”. Self-efficacy scale ranging from not at all confident “1” to very confident “4”. - The TPB construct, subjective norms, and behavioral control were each assessed through 1 item. With scoring using a 4-point scale that ranged from never “1” to often “4”. - MMG screening was ascertained through self-report + medical records.	Content validity was conducted by panel experts Reliability: - The HBM constructs: Cronbach’s alpha for susceptibility = 0.84, severity = 0.82, benefits of MMG = 0.72, barrier = 0.73, self-efficacy = 0.90. - The TPB constructs: The test-retest reliability coefficient was 0.84 for subjective norms and 0.87 for perceived behavioral control.
Rabbani et al., 2019 [61] - BC knowledge	- Baseline - 4 weeks after the intervention	- Face-to-face questionnaire. Adapted questionnaire consisted of 3 questions that assessed general knowledge of BC, 6 questions on BC symptoms, 2 questions on knowledge of age-related and BC lifetime risks, 8 questions on BC risk factors, 4 questions on BC awareness, 2 questions on BC treatment, 2 questions on skills, behavior, and confidence, 4 questions on barriers of seeking medical help.	Psychometric properties were not reported.
Gondek et al., 2015 [58] - BC knowledge - MMG screening	- Knowledge was assessed during a single session (pre–post test) - MMG was assessed at: -Baseline - 2 years after the intervention	- An audience response system (ARS) or paper surveys + MMG records. - Participants received an audience answer technique or paper surveys to deliver their replies regarding BCS. - 6 items assessed knowledge. Details of the items were not stated.	Psychometric properties were not reported.
Ouyang and Hu, 2014 [62] - BSE practice - Knowledge of BC - HBM scales	- Baseline - 1 month after the intervention - 3 months after the intervention	- SAQ. - A 14-item checklist used to assess BSE, with yes and no answers. Scores range from 0–14. - 17 items assessed knowledge in 3 domains, BC symptoms, BC risk factors, and early screening approaches, with yes and no answer. Right answer (1 point) and incorrect answer (0 point). - HBM scales: 35 items on susceptibility, seriousness, BSE benefits and barriers, and confidence, with a 5-point Likert scale that range from disagrees to agree.	- BSE checklist Cronbach’s alpha coefficient = 0.89. - Knowledge questionnaire Cronbach’s alpha = 0.54. - HBM scales Cronbach’s alpha coefficients for susceptibility = 0.78, seriousness = 0.68, benefits = 0.63, barriers = 0.74, confidence = 0.89.
Seven et al., 2014 [60] - MMG performance - Knowledge - HBM scales	- Baseline - 3 months after the intervention	- SAQ. - 5 items reason identification questionnaire (RIF) used to assess factors that effect a participant’s decision to get screening, and to evaluate satisfaction with screening practice. - (PDQ) questionnaire that included items on Knowledge Evaluation Form (KEF): included 15 positive statements on knowledge on BC, BSE, and CBE and the proper time of getting MMG. With true or false choices ranging from 0–15. - CHBMS consists of 3 items on susceptibility, 6 items on seriousness, 5 items on health motivation, 5 items on MMG benefits, and 11 items on MMG barriers. With a 5-point Likert scale that ranged from 1–5.	- Cronbach’s alpha coefficient for the KEF = 0.86. - Cronbach’s alpha coefficients for susceptibility = 0.99, seriousness = 0.8, health motivation = 0.94, benefits = 0.61, barriers = 0.86.
Tuzcu et al., 2016 [10] - BSE practice - CBE practice - MMG practice - HBM scales	- Baseline - 3 months after the intervention - 6 months after the intervention	- A question/answer method through face-to-face interviews, performing BCS through a telephone interview with 3 questions on the practice of BSE, CBE, and MMG. - The screening behaviors status were assessed through a telephone interview at month 3, and by using a structured questionnaire at month 6. - The CHBMS used to assess health beliefs using 3 items on susceptibility, 6 items on seriousness, 5 items on health motivation, 4 items on BSE benefits, 8 items on BSE barriers, 10 items on self-efficacy, 5 items on MMG benefits, 11 items on MMG barriers. With 5-point Likert options. - Health responsibility was assessed using the Turkish version of Healthy Lifestyle Behaviors Scale II, with scores that ranged from 9–36.	- CHBMS Cronbach’s alpha coefficients were between 0.61 and 0.71. - Health responsibility Cronbach’s alpha coefficient = 0.70
Vasishta et al., 2018 [63] - Knowledge about BC and BSE	- Baseline - Post-intervention	- SAQ in English language. - 20 MCQs measure BC and BSE knowledge and awareness. Scores were classified as poor from 0 to 7, average from 8 to 12, and good from 13 and above.	Tests of validity and reliability were conducted, but details of the psychometric properties were not reported in the article.
Lee-Lin et al., 2015a [57] - MMG screening	- Baseline - 3 months after the intervention - 6 months after the intervention - 12 months after the intervention	Self-report questionnaire.	
Wu and Lin, 2016 [56] - MMG screening	- Baseline - 4 months after the intervention	Self-report questionnaire.	

## Data Availability

Data is contained within the Supplementary Materials.

## Supplementary Materials

The following are available online at https://www.mdpi.com/1660-4601/18/1/263/s1. Table S1: PRISMA checklist; Table S2: Search strategy.
