# Supplementary material for: The Effectiveness of Educational Interventions on Breast Cancer Screening Uptake, Knowledge, and Beliefs among Women: A Systematic Review
Source: Int J Environ Res Public Health. 2020 Dec 31;18(1):263. doi: 10.3390/ijerph18010263 (PMC7795851; doi:10.3390/ijerph18010263)

S3 File

Search strategy for PubMed - May 11, 2019

NCBI Resources How To Sign in to NCBI

PubMed.gov
US National Library of Medicine
National Institutes of Health

PubMed | (((("breast cancer") AND (("early detection" OR "early diagnosis" OR screening OR practice OR be | Search

Advanced Help

Filters activated: Publication date from 2014/01/01 to 2019/05/31, Humans, English. Clear all

Search Details

Query Translation:

```
(("breast cancer"[All Fields] AND ("early detection"[All Fields]
OR "early diagnosis"[All Fields] OR ("diagnosis"[Subheading] OR
"diagnosis"[All Fields] OR "screening"[All Fields] OR "mass
screening"[MeSH Terms] OR ("mass"[All Fields] AND "screening"
[All Fields]) OR "mass screening"[All Fields] OR "screening"[All
Fields] OR "early detection of cancer"[MeSH Terms] OR ("early"
[All Fields] AND "detection"[All Fields] AND "cancer"[All
Fields]) OR "early detection of cancer"[All Fields]) OR
("Practice (Birm)"[Journal] OR "practice"[All Fields]) OR
("behaviour"[All Fields] OR "behavior"[MeSH Terms] OR "behavio
```

Search URL

Result:

157

Translations:

screening	"diagnosis"[Subheading] OR "diagnosis"[All Fields] OR "screening"[All Fields] OR "mass screening"[MeSH Terms] OR ("mass"[All Fields] AND "screening"[All Fields]) OR "mass screening"[All Fields] OR "screening"[All Fields] OR "early detection of cancer"[MeSH Terms] OR ("early"[All Fields] AND "detection"[All Fields] AND "cancer"[All Fields]) OR "early detection of cancer"[All Fields]
practice	"Practice (Birm)"[Journal] OR "practice"[All Fields]
behaviour	"behaviour"[All Fields] OR "behavior"[MeSH Terms] OR "behavior"[All Fields]
Health education	"health education"[MeSH Terms] OR ("health"[All Fields] AND "education"[All Fields]) OR "health education"[All Fields]
intervention	"methods"[MeSH Terms] OR "methods"[All Fields] OR "intervention"[All Fields]
[illegible]	[illegible]

History

Download history Clear history

Search	Add to builder	Query	Items found	Time
#14	Add	Search **(((("breast cancer") AND ((("early detection" OR "early diagnosis" OR screening OR practice OR behavior)))) AND ((Health education OR module OR program OR intervention))) AND ((knowledge OR awareness))) AND beliefs** Filters: **Publication date from 2014/01/01 to 2019/05/31; Humans; English**	157	23:33:01
#13	Add	Search **(((("breast cancer") AND ((("early detection" OR "early diagnosis" OR screening OR practice OR behavior)))) AND ((Health education OR module OR program OR intervention))) AND ((knowledge OR awareness))) AND beliefs** Filters: **Humans; English**	531	23:20:01
#12	Add	Search **(((("breast cancer") AND ((("early detection" OR "early diagnosis" OR screening OR practice OR behavior)))) AND ((Health education OR module OR program OR intervention))) AND ((knowledge OR awareness))) AND beliefs** Filters: **Humans**	538	23:19:48
#11	Add	Search **(((("breast cancer") AND ((("early detection" OR "early diagnosis" OR screening OR practice OR behavior)))) AND ((Health education OR module OR program OR intervention))) AND ((knowledge OR awareness))) AND beliefs**	596	23:19:26
#10	Add	Search **("culture"[MeSH Terms] OR "culture"[All Fields] OR "beliefs"[All Fields]) OR (("health"[MeSH Terms] OR "health"[All Fields]) AND ("culture"[MeSH Terms] OR "culture"[All Fields] OR "beliefs"[All Fields]))**	947250	23:11:37
#9	Add	Search **(beliefs OR health beliefs)**	947250	23:11:29
#8	Add	Search **beliefs**	947250	23:10:44
#7	Add	Search **(knowledge OR awareness)**	813476	23:10:07
#6	Add	Search **knowledge**	703455	23:09:29
#5	Add	Search **(Health education OR module OR program OR intervention)**	8334957	23:06:12
#4	Add	Search **(("early detection" OR "early diagnosis" OR screening OR practice OR behavior))**	7165299	23:03:14
#3	Add	Search **((early detection OR early diagnosis OR screening OR practice OR behavior))**	7182341	23:00:58
#2	Add	Search **"breast cancer"**	254001	22:58:12

Search strategy for Science Direct - May 14, 2019

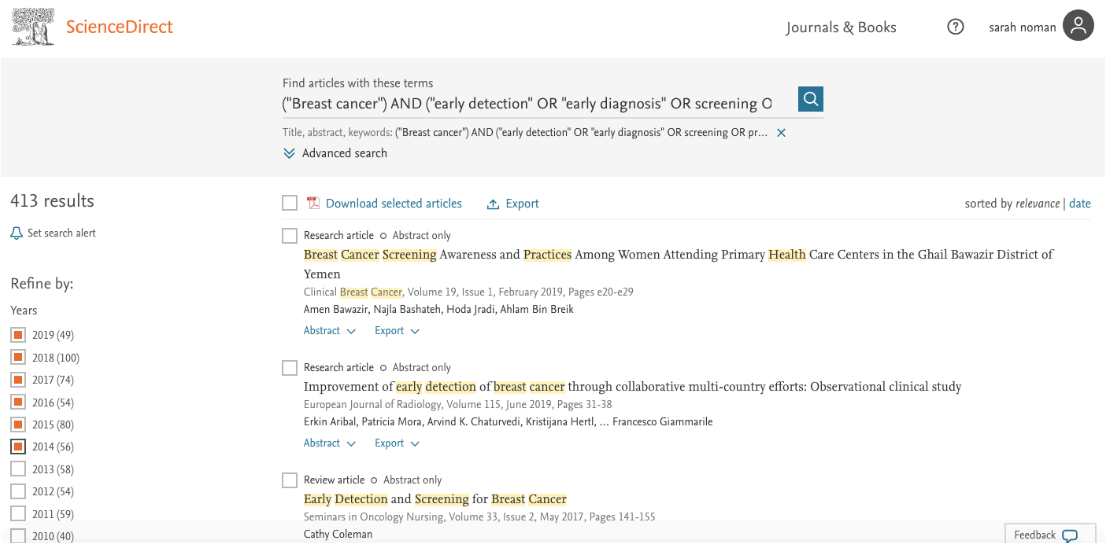

Search strategy for Scopus - May 14, 2019

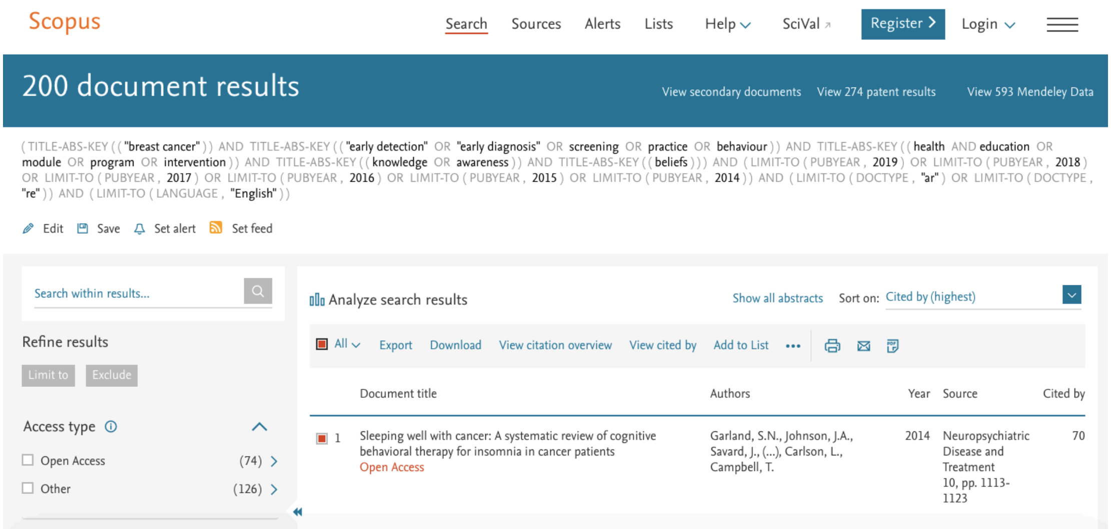

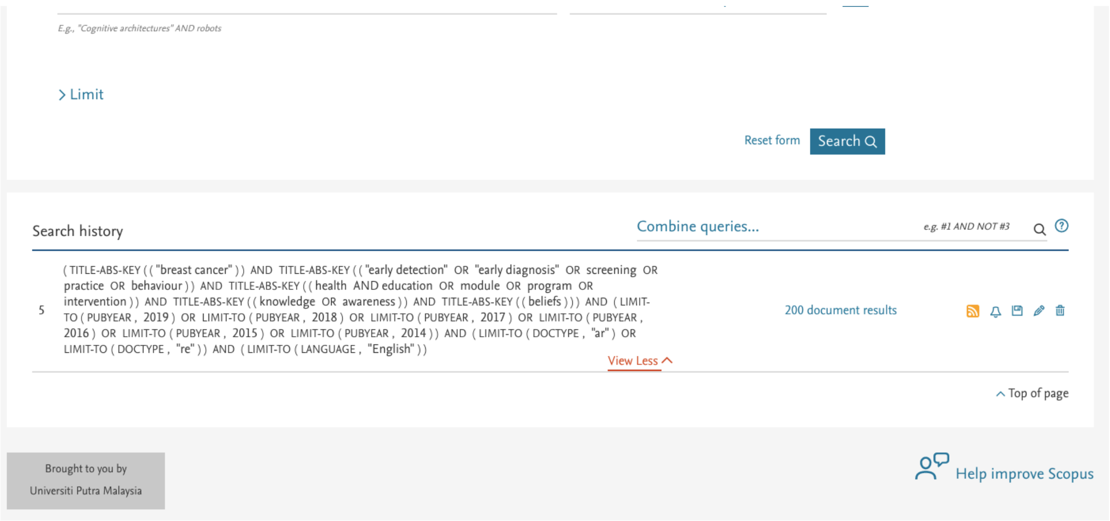

Search strategy for Web of Science - May 13, 2019

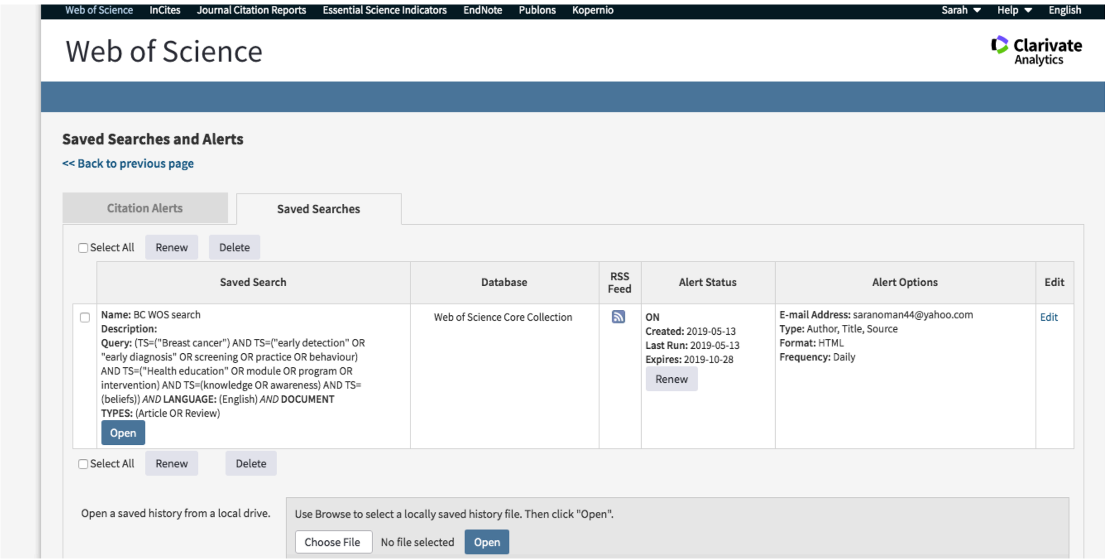

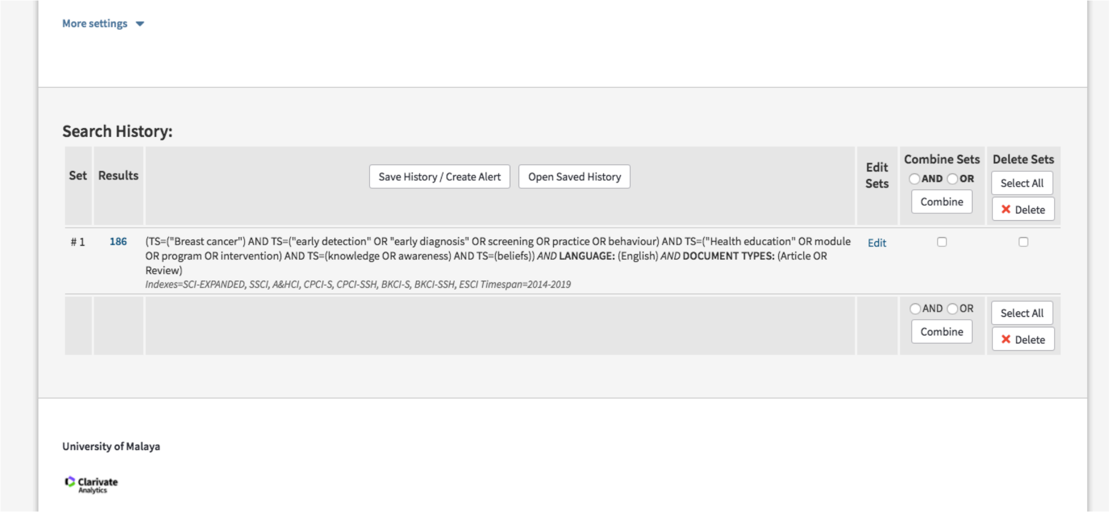

Supplement: Supplementary file 1 [file ijerph-18-00263-s001.zip › S2 File.pdf]
